# Supplementary material for: Learning gene networks underlying clinical phenotypes using SNP perturbation
Source: PLoS Comput Biol. 2020 Oct 23;16(10):e1007940. doi: 10.1371/journal.pcbi.1007940 (PMC7584257; doi:10.1371/journal.pcbi.1007940)
Supplement: S1 Text — (PDF) [file pcbi.1007940.s001.pdf]

S1 Text. Two-layer Lasso

We compare the performance of our method with that of Lasso, a popular statistical method based on linear regression models for studying associations among SNPs, expression measurements, and phenotypes. We begin by setting up a two-layer multivariate regression model for genotypes $\mathbf{x} \in \{0,1,2\}^p$, expression measurements $\mathbf{y} \in \mathbb{R}^q$, and phenotypes $\mathbf{z} \in \mathbb{R}^r$ as follows:

$$\mathbf{y} = \mathbf{A}_{\mathbf{xy}}^T \mathbf{x} + \epsilon_{\mathbf{y}}, \quad \epsilon_{\mathbf{y}} \sim \mathcal{N}(\mathbf{0}_q, \mathbf{\Omega}_{\mathbf{y}}),$$
$$\mathbf{z} = \mathbf{A}_{\mathbf{yz}}^T \mathbf{y} + \epsilon_{\mathbf{z}}, \quad \epsilon_{\mathbf{z}} \sim \mathcal{N}(\mathbf{0}_r, \mathbf{\Omega}_{\mathbf{z}}),$$

where $\mathbf{A}_{\mathbf{xy}} \in \mathbb{R}^{p\times q}$ and $\mathbf{A}_{\mathbf{yz}} \in \mathbb{R}^{q\times r}$ are regression coefficients, $\epsilon_{\mathbf{y}} \in \mathbb{R}^q$ and $\epsilon_{\mathbf{z}} \in \mathbb{R}^r$ are noise distributed with zero means and diagonal covariances $\mathbf{\Omega}_{\mathbf{y}} = \mathrm{diag}(\sigma^2_{\mathbf{y}_1}, \ldots, \sigma^2_{\mathbf{y}_q})$ and $\mathbf{\Omega}_{\mathbf{z}} = \mathrm{diag}(\sigma^2_{\mathbf{z}_1}, \ldots, \sigma^2_{\mathbf{z}_q})$.

Given genotype data $\mathbf{X} \in \{0,1,2\}^{n\times p}$ for n samples and p SNPs, expression data $\mathbf{Y} \in \mathbb{R}^{n\times q}$ for q genes, and phenotype data $\mathbf{Z} \in \mathbb{R}^{n\times r}$ for r phenotypes, we obtain a Lasso estimate of the regression coefficients by minimizing L_1-regularized negative log-likelihood as follows:

$$\min_{\mathbf{A}_{\mathbf{xy}}, \mathbf{A}_{\mathbf{yz}}} \frac{1}{n} \mathrm{tr}\left(\left(\mathbf{Y} - \mathbf{X}\mathbf{A}_{\mathbf{xy}}\right)\left(\mathbf{Y} - \mathbf{X}\mathbf{A}_{\mathbf{xy}}\right)^T + \left(\mathbf{Z} - \mathbf{Y}\mathbf{A}_{\mathbf{yz}}\right)\left(\mathbf{Z} - \mathbf{Y}\mathbf{A}_{\mathbf{yz}}\right)^T \right)$$
$$+\gamma_1 ||\mathbf{A}_{\mathbf{xy}}||_1 + \gamma_2 ||\mathbf{A}_{\mathbf{yz}}||_1.$$

We select the regularization parameters γ_1 and γ_2 using BIC.
