# Supplementary material for: Learning gene networks underlying clinical phenotypes using SNP perturbation
Source: PLoS Comput Biol. 2020 Oct 23;16(10):e1007940. doi: 10.1371/journal.pcbi.1007940 (PMC7584257; doi:10.1371/journal.pcbi.1007940)
Supplement: S2 Text — (PDF) [file pcbi.1007940.s002.pdf]

S2 Text. Fast-sCGGM and Mega-sCGGM for efficient sCGGM optimization

Fast-sCGGM pseudocode. The pseudocode for Fast-sCGGM is provided in Algorithm 1.

Algorithm 1: Fast-sCGGM

input : Inputs $\mathbf{X} \in \mathbb{R}^{n \times p}$ and $\mathbf{Y} \in \mathbb{R}^{n \times q}$; regularization parameters $\lambda_{\mathbf{\Lambda}}, \lambda_{\mathbf{\Theta}}$
output : Parameters $\mathbf{\Lambda}, \mathbf{\Theta}$
Initialize $\mathbf{\Theta} \leftarrow 0, \mathbf{\Lambda} \leftarrow I_q$
for $t = 0, 1, \ldots$ **do**
  Determine active sets $\mathcal{S}_{\mathbf{\Lambda}}, \mathcal{S}_{\mathbf{\Theta}}$
  Solve via coordinate descent: $D_{\mathbf{\Lambda}} = \arg\min_{\Delta_{\mathbf{\Lambda}}, \Delta_{\overline{\mathcal{S}_{\mathbf{\Lambda}}}=0}} \bar{g}_{\mathbf{\Lambda},\mathbf{\Theta}}(\mathbf{\Lambda} + \Delta_{\mathbf{\Lambda}}, \mathbf{\Theta}) + h(\mathbf{\Lambda} + \Delta_{\mathbf{\Lambda}}, \mathbf{\Theta})$
  Update $\mathbf{\Lambda} = \mathbf{\Lambda} + \alpha D_{\mathbf{\Lambda}}$, where step size α is found with line search
  Solve via coordinate descent: $\mathbf{\Theta} = \mathrm{argmin}_{\mathbf{\Theta}_{\mathcal{S}_{\mathbf{\Theta}}}} \; g_{\mathbf{\Lambda}}(\mathbf{\Theta}) + \lambda_{\mathbf{\Theta}} \|\mathbf{\Theta}\|_1$

Mega-sCGGM for removing memory constraint. We provide details of the block-wise update in the coordinate descent optimization for $\mathbf{\Lambda}$ and $\mathbf{\Theta}$ in Mega-sCGGM.

Blockwise optimization for $\mathbf{\Lambda}$. A coordinate-descent update of $[\Delta_{\mathbf{\Lambda}}]_{i,j}$ requires the ith and jth columns of $\mathbf{\Sigma}$ and $\mathbf{\Psi}$. If these columns are in memory, they can be re-used. Otherwise, it is a cache miss and we should compute them on demand as follows. We obtain $[\mathbf{\Sigma}]_{:,i}$ by solving linear system $\mathbf{\Lambda}[\mathbf{\Sigma}]_{:,i} = \mathbf{e}_i$, where $\mathbf{e}_i$ is a vector of q 0's except for 1 in the ith element, with conjugate gradient method. Then, $[\mathbf{\Psi}]_{:,i}$ can be obtained from $\mathbf{R}^T[\mathbf{R}]_{:,i}$, where $\mathbf{R} = \mathbf{X}\mathbf{\Theta}\mathbf{\Sigma}$.

In order to reduce cache misses, we perform block coordinate descent, where within each block, the columns of $\mathbf{\Sigma}$ are cached and re-used. Suppose we partition $\mathcal{N} = \{1, \ldots, q\}$ into $k_{\mathbf{\Lambda}}$ disjoint sets, $C_1, \ldots, C_{k_{\mathbf{\Lambda}}}$. We apply this partitioning to the rows and columns of $\Delta_{\mathbf{\Lambda}}$ to obtain $k_{\mathbf{\Lambda}} \times k_{\mathbf{\Lambda}}$ blocks. Then, we perform coordinate-descent updates in each block, updating all elements in the active set within that block. Let $[\mathbf{A}]_{:,C_r}$ denote a submatrix of $\mathbf{A}$ with columns in partition C_r. In order to perform coordinate-descent updates on (C_r, C_z) block of $\Delta_{\mathbf{\Lambda}}$, we need $[\mathbf{\Sigma}]_{:,C_r}$, $[\mathbf{\Sigma}]_{:,C_z}$, $[\mathbf{\Psi}]_{:,C_r}$, and $[\mathbf{\Psi}]_{:,C_z}$. Thus, we pick the smallest possible $k_{\mathbf{\Lambda}}$ such that we can store $q/k_{\mathbf{\Lambda}}$ columns of $\mathbf{\Sigma}$ and $\mathbf{\Psi}$ in memory. When updating the variables within block (C_z, C_r) of $\Delta_{\mathbf{\Lambda}}$, there are no cache misses once $[\mathbf{\Sigma}]_{:,C_r}$, $[\mathbf{\Sigma}]_{:,C_z}$, $[\mathbf{\Psi}]_{:,C_r}$, and $[\mathbf{\Psi}]_{:,C_z}$ are computed and stored. After updating each $[\Delta_{\mathbf{\Lambda}}]_{i,j}$ to $[\Delta_{\mathbf{\Lambda}}]_{i,j} + \mu$, we maintain $[\mathbf{U}]_{:,C_z}$ and $[\mathbf{U}]_{:,C_r}$ by computing $[\mathbf{U}]_{i,t} \leftarrow [\mathbf{U}]_{i,t} + \mu[\mathbf{\Sigma}]_{j,t}$ and $[\mathbf{U}]_{j,t} \leftarrow [\mathbf{U}]_{j,t} + \mu[\mathbf{\Sigma}]_{i,t}, \forall t \in \{C_z \cup C_r\}$.

To go through all blocks, for each $z \in \{1, \ldots, k_{\mathbf{\Lambda}}\}$, we update blocks $(C_z, C_1), \ldots, (C_z, C_k)$. Since all of these blocks for the given z require $[\mathbf{\Sigma}]_{:,C_z}$ and $[\mathbf{\Psi}]_{:,C_z}$ for coordinate descent update, we precompute and store them in memory. When updating an off-diagonal block $(C_z, C_r), z \neq r$, we precompute $[\mathbf{\Sigma}]_{:,C_r}$ and $[\mathbf{\Psi}]_{:,C_r}$.

In typical gene or trait networks, the graph structure of $\mathbf{\Lambda}$ will exhibit clustering, with an approximately block diagonal structure. We exploit this structure by choosing a partition $\{C_1, \ldots, C_{k_{\mathbf{\Lambda}}}\}$ that minimizes cache misses. Within diagonal blocks (C_r, C_r)'s, once $[\mathbf{\Sigma}]_{:,C_r}$ and $[\mathbf{\Psi}]_{:,C_r}$ are computed, there are no cache misses. For off-diagonal blocks (C_r, C_z)'s, $r \neq z$, we have a cache miss only if some variable in $\{[\Delta]_{i,j} | i \in C_r, j \in C_z\}$ lies in the active set. We minimize the active set in off-diagonal blocks via clustering, following the strategy for sparse GGM estimation in BIG&QUIC and using the METIS graph clustering library.

Although the worst-case scenario is to compute $\mathbf{\Sigma}$ and $\mathbf{\Psi}$ $k_{\mathbf{\Lambda}}$ times to update all elements of $\Delta_{\mathbf{\Lambda}}$, in practice, graph clustering dramatically reduces this cost. In the best case, if the active set for $\mathbf{\Lambda}$ is perfectly block-diagonal and graph clustering identifies this block diagonal structure, we need to compute $\mathbf{\Sigma}$ and $\mathbf{\Psi}$ only once to update all the blocks. A depiction of our blockwise optimization scheme is given in S8A Fig.

Blockwise Optimization for $\mathbf{\Theta}$. The coordinate descent update of $[\mathbf{\Theta}]_{i,j}$ requires $[\mathbf{S}_{\mathbf{xx}}]_{:,i}$ and $[\mathbf{\Sigma}]_{:,j}$ to compute $[\mathbf{S}_{\mathbf{xx}}]_{:,i}^T[\mathbf{V}]_{:,j}$, where $[\mathbf{V}]_{:,j} = \mathbf{\Theta}[\mathbf{\Sigma}]_{:,j}$. If $[\mathbf{S}_{\mathbf{xx}}]_{:,i}$ and $[\mathbf{\Sigma}]_{:,j}$ are not already in the memory, it is a cache miss. Computing $[\mathbf{S}_{\mathbf{xx}}]_{:,i}$ takes $O(np)$, which is expensive if we have many cache misses.

We propose a block coordinate descent approach for updating $\mathbf{\Theta}$ that groups these computations to reduce cache misses. Given a partition of $\{1, \ldots, q\}$ into $k_{\mathbf{\Theta}}$ subsets, $C_1, \ldots, C_{k_{\mathbf{\Theta}}}$, we divide $\mathbf{\Theta}$ into $p \times k_{\mathbf{\Theta}}$ blocks, where each block comprises a portion of a row of $\mathbf{\Theta}$. We denote each block (i, C_r), where $i \in \{1, \ldots, p\}$ and $C_r \in \{C_1, \ldots, C_{k_{\mathbf{\Theta}}}\}$. Since updating block (i, C_r) requires $[\mathbf{S}_{\mathbf{xx}}]_{:,i}$ and $[\mathbf{\Sigma}]_{:,C_r}$, we select the smallest possible $k_{\mathbf{\Theta}}$ such that we can store $q/k_{\mathbf{\Theta}}$ columns of $\mathbf{\Sigma}$ in memory. While performing coordinate descent updates within block (i, C_r) of $\mathbf{\Theta}$, there are no cache misses, once $[\mathbf{S}_{\mathbf{xx}}]_{:,i}$ and $[\mathbf{\Sigma}]_{:,C_r}$ are in memory. After updating each $[\mathbf{\Theta}]_{i,j}$ to $[\mathbf{\Theta}]_{i,j} + \mu$, we update $[\mathbf{V}]_{:,C_r}$ by $[\mathbf{V}]_{i,t} \leftarrow [\mathbf{V}]_{i,t} + \mu[\mathbf{\Sigma}]_{j,t}, \forall t \in C_r$.

Algorithm 2: Mega-sCGGM

```
input  : X ∈ ℝ^{n×p} and Y ∈ ℝ^{n×q}; regularization parameters λ_Λ, λ_Θ
output : Parameters Λ, Θ
Initialize Θ ← 0, Λ ← I_q
for t = 0, 1, ... do
    Determine active sets S_Λ, S_Θ
    Partition columns of Λ into k_Λ blocks                          ▷ Minimize over Λ
    Initialize Δ_Λ ← 0
    for z = 1 to k_Λ do
        Compute [Σ]_{:,C_z}, [U]_{:,C_z}, and [Ψ]_{:,C_z}
        for r = 1 to k_Λ do
            if z ≠ r then
                Identify columns B_{zr} ⊂ C_r with active elements in Λ
                Compute [Σ]_{:,B_{zr}}, [U]_{:,B_{zr}}, and [Ψ]_{:,B_{zr}}
            Update all active [Δ_Λ]_{i,j} in (C_z, C_r)
    Update Λ ← Λ + αΔ_Λ, where step size α is found by line search
    Partition columns of Θ into k_Θ blocks                          ▷ Minimize over Θ
    for r = 1 to k_Θ do
        Compute [Σ]_{:,C_r}, and initialize V ← Θ[Σ]_{:,C_r}
        for row i ∈ {1, ..., p} if I_φ(S_{(i,C_r)}) do
            Compute [S_xx]_{i,j} for non-empty columns j in V_{C_r}
            Update all active [Θ]_{i,j} in (i, C_r)
```

In order to sweep through all blocks, for each $r \in \{1, \ldots, k_{\mathbf{\Theta}}\}$ we update blocks $(1, C_r), \ldots, (p, C_r)$. Since all of these p blocks with the same C_r share the computation of $[\mathbf{\Sigma}]_{:,C_r}$, we compute and store $[\mathbf{\Sigma}]_{:,C_r}$ in memory. Within each block, the computation of $[\mathbf{S_{xx}}]_{:,i}$ is shared, so we precompute and store it in memory, before updating this block. The full matrix of $\mathbf{\Sigma}$ will be computed once while sweeping through the full $\mathbf{\Theta}$, whereas $\mathbf{S_{xx}}$ will be computed $k_{\mathbf{\Theta}}$ times.

We further reduce cache misses for $[\mathbf{S_{xx}}]_{:,i}$ by strategically selecting partition $C_1, \ldots, C_{k_{\mathbf{\Theta}}}$, based on the observation that if the active set is empty in block (i, C_r), we can skip this block and forgo computing $[\mathbf{S_{xx}}]_{:,i}$. We therefore choose a partition where the active set variables are clustered into as few blocks as possible. Formally, we want to minimize $\sum_{i,q} |I_\phi(\mathcal{S}_{(i,C_r)})|$, where $I_\phi(\mathcal{S}_{(i,C_r)})$ is an indicator function that outputs 1 if the active set $\mathcal{S}_{(i,C_r)}$ within block (i, C_r) is not empty and 0 otherwise. We perform graph clustering over the graph $G = (V, E)$ defined from the active set in $\mathbf{\Theta}$, where $V = \{1, \ldots, q\}$ and $E = \{(j,k) | \exists i \in \{1, ..., p\} \ [\mathbf{\Theta}]_{i,j} \in \mathcal{S}_{\mathbf{\Theta}}, [\mathbf{\Theta}]_{i,k} \in \mathcal{S}_{\mathbf{\Theta}}\}$, connecting two nodes j and k with an edge if both $[\mathbf{\Theta}]_{i,j}$ and $[\mathbf{\Theta}]_{i,k}$ are in the active set. This edge set corresponds to the non-zero elements of $\mathbf{\Theta}^T\mathbf{\Theta}$, so the graph can be computed efficiently.

We also exploit row-wise sparsity in $\mathbf{\Theta}$ to reduce the cost of each cache miss. Every empty row in $\mathbf{\Theta}$ corresponds to an empty row in $\mathbf{V} = \mathbf{\Theta}\mathbf{\Sigma}$. Because we only need elements in $[\mathbf{S_{xx}}]_{:,i}$ for the dot product $[\mathbf{S_{xx}}]_{:,i}^T [\mathbf{V}]_{:,j}$, we skip computing the kth element of $[\mathbf{S_{xx}}]_{:,i}$ if the kth row of $\mathbf{\Theta}$ is all zeros. Our blockwise optimization scheme for $\mathbf{\Theta}$ is depicted in S8B Fig.
