# Supplementary figures and images for: Learning gene networks underlying clinical phenotypes using SNP perturbation
Source: PLoS Comput Biol. 2020 Oct 23;16(10):e1007940. doi: 10.1371/journal.pcbi.1007940 (PMC7584257; doi:10.1371/journal.pcbi.1007940)

**A**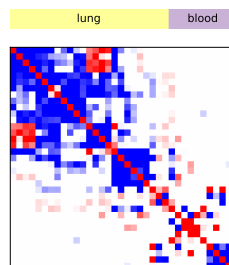**C**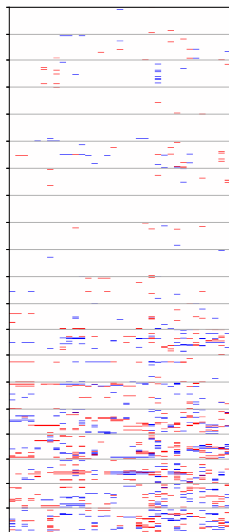**B**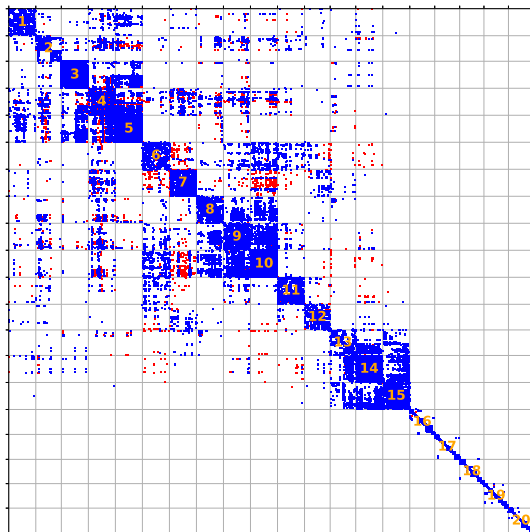**D**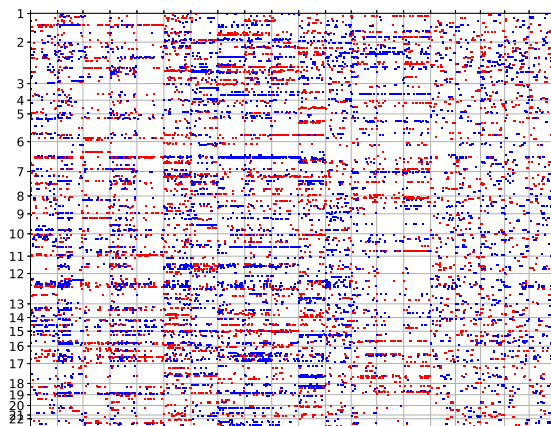

Supplement: S1 Fig — (PDF) [file pcbi.1007940.s003.pdf]

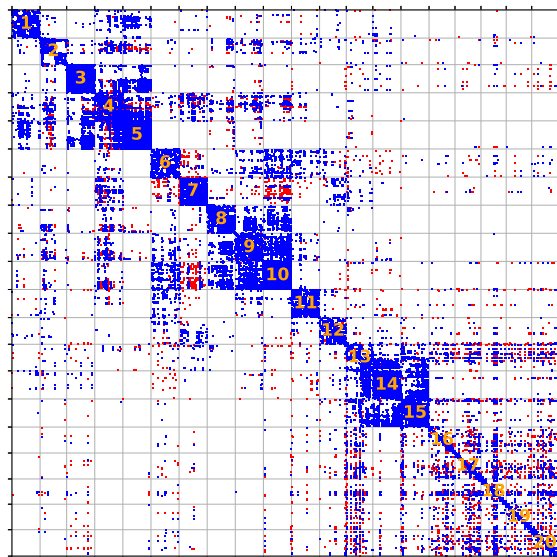

Supplement: S2 Fig — (PDF) [file pcbi.1007940.s004.pdf]

**A**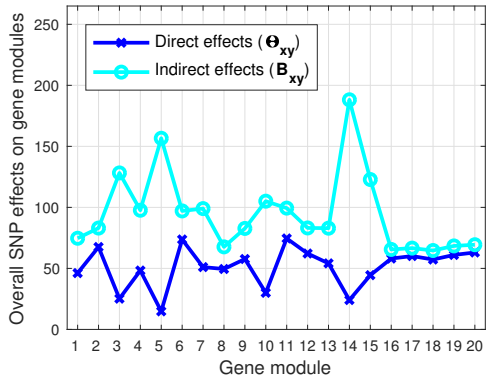**B**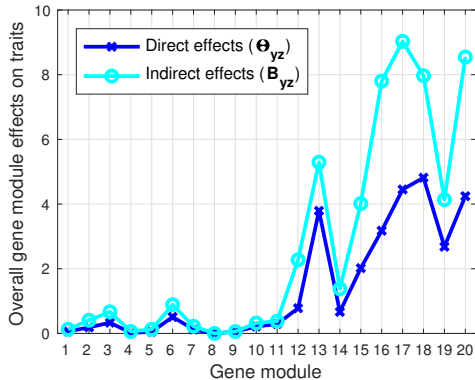

Supplement: S3 Fig — (PDF) [file pcbi.1007940.s005.pdf]

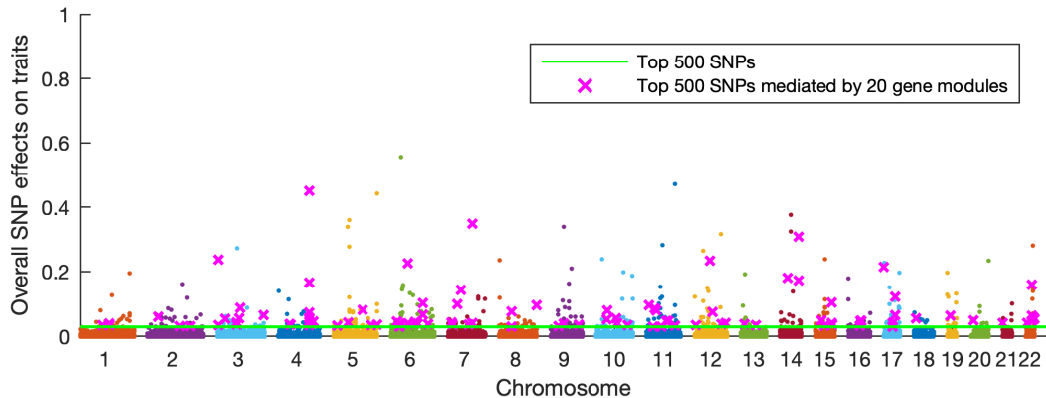

Supplement: S4 Fig — (PDF) [file pcbi.1007940.s006.pdf]

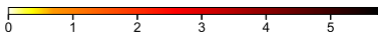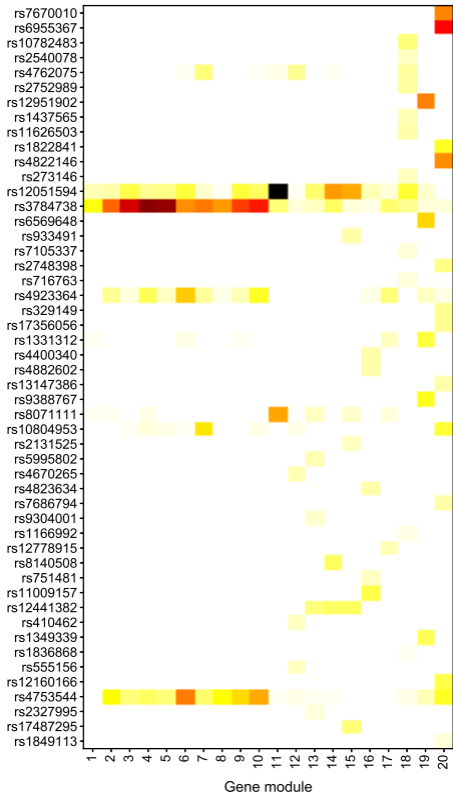

Supplement: S5 Fig — (PDF) [file pcbi.1007940.s007.pdf]

**A**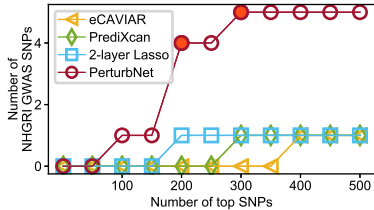**B**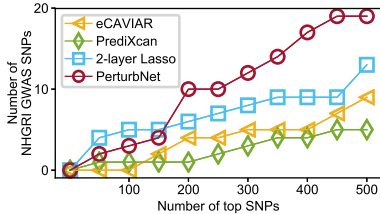**C**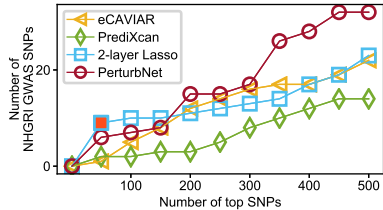

Supplement: S6 Fig — (PDF) [file pcbi.1007940.s008.pdf]

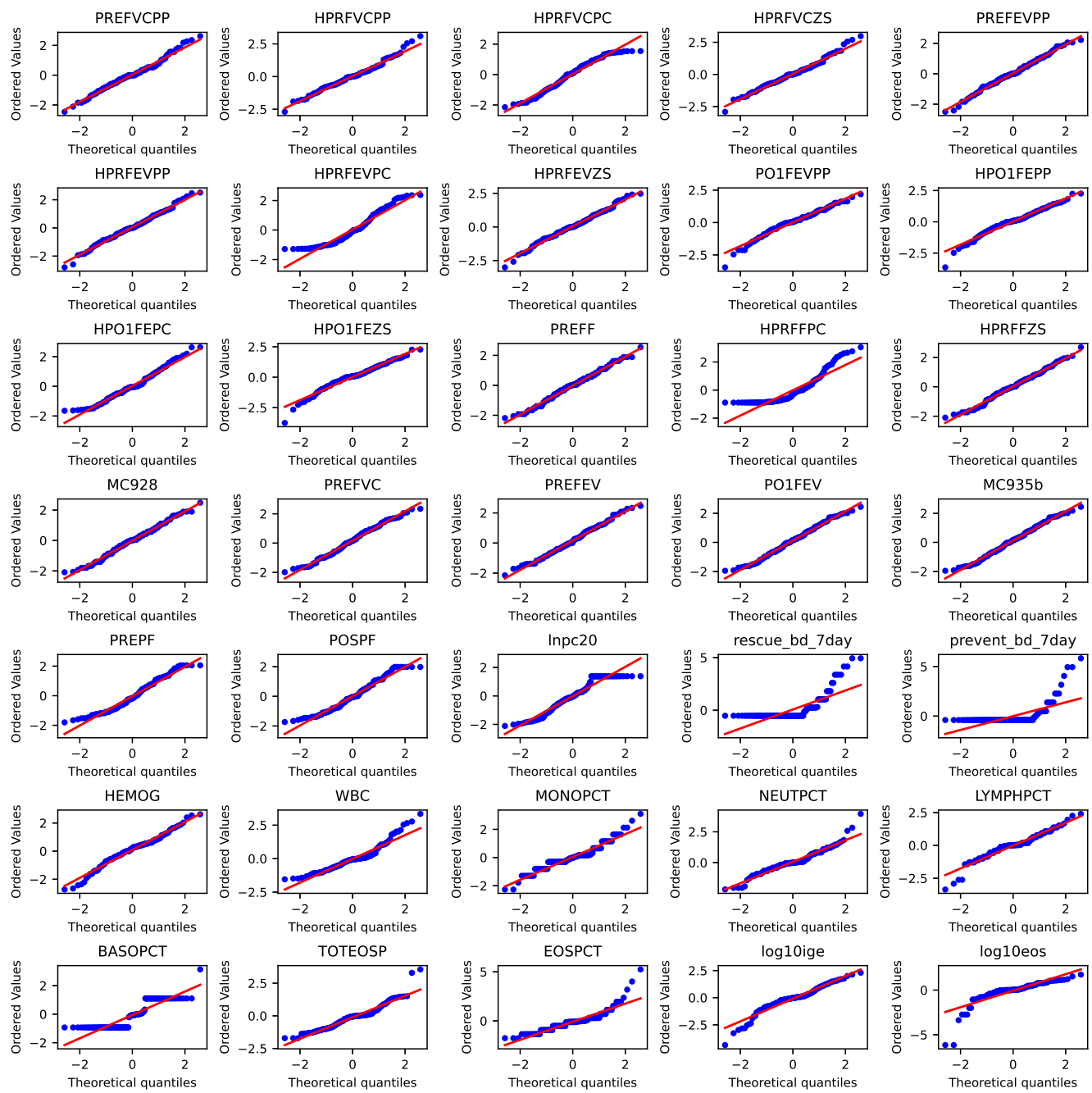

Supplement: S7 Fig — (PDF) [file pcbi.1007940.s009.pdf]

A

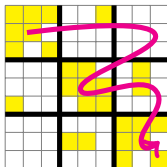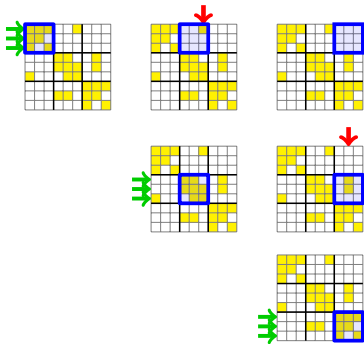

B

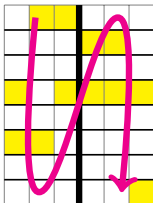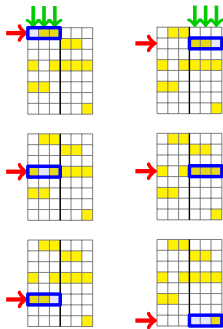

Supplement: S8 Fig — (PDF) [file pcbi.1007940.s010.pdf]
