# Supplementary material for: Learning gene networks underlying clinical phenotypes using SNP perturbation
Source: PLoS Comput Biol. 2020 Oct 23;16(10):e1007940. doi: 10.1371/journal.pcbi.1007940 (PMC7584257; doi:10.1371/journal.pcbi.1007940)
Supplement: S1 Table — (PDF) [file pcbi.1007940.s011.pdf]

Module	Size	GO terms in biological processes	p-value	Overlap[†]
1	314	Cellular macromolecule metabolic process	2.94×10^{-12}	159 / 7006
2	297	Cellular nitrogen compound metabolic process	1.64×10^{-4}	112 / 5164
3	314	Nucleobase-containing compound metabolic process	5.62×10^{-13}	123 / 4538
4	314	Organelle organization	2.02×10^{-4}	79 / 3167
5	314	Nucleobase-containing compound metabolic process	4.58×10^{-4}	106 / 4538
6	314	Unclassified	NA	NA
7	314	Cellular localization	1.80×10^{-4}	60 / 2287
8	314	Cellular metabolic process	1.73×10^{-4}	178 / 9003
9	314	Cellular metabolic process	8.39×10^{-6}	185 / 9003
10	314	Macromolecule metabolic process	8.73×10^{-5}	159 / 7749
11	314	Heterocycle metabolic process	7.63×10^{-3}	103 / 4715
12	298	Translation	1.65×10^{-9}	27 / 383
**13**[‡]	297	Immune system process	3.66×10^{-12}	83 / 2552
		Response to stimulus	1.70×10^{-9}	162 / 8009
		Response to stress	8.43×10^{-9}	90 / 3333
**14**[‡]	314	Immune response	3.96×10^{-38}	106 / 1673
		Leukocyte activation involved in immune response	9.04×10^{-31}	62 / 607
		Granulocyte activation	1.24×10^{-26}	53 / 495
**15**[‡]	313	Cell activation in immune response	4.95×10^{-32}	65 / 611
		Myeloid leukocyte activation	5.28×10^{-32}	63 / 566
		Immune system process	1.01×10^{-31}	124 / 2552
16	290	Cellular process	3.73×10^{-3}	132 / 15013
17	290	Regulation of macromolecule metabolic process	1.58×10^{-3}	74 / 6142
18	282	Organonitrogen compound metabolic process	1.97×10^{-2}	60 / 5523
19	285	Cell cycle	1.50×10^{-8}	37 / 1355
20	295	Cellular component organization or biogenesis	4.34×10^{-3}	66 / 5525

[†] The number of genes in the overlap / the total number of genes in the GO category

[‡] Asthma gene modules enriched with genes in immune system GO categories
