# Supplementary material for: Learning gene networks underlying clinical phenotypes using SNP perturbation
Source: PLoS Comput Biol. 2020 Oct 23;16(10):e1007940. doi: 10.1371/journal.pcbi.1007940 (PMC7584257; doi:10.1371/journal.pcbi.1007940)
Supplement: S2 Table — (PDF) [file pcbi.1007940.s012.pdf]

Size	GO terms in biological processes	p-value	Overlap†
374	Cellular response to stress	2.90 $\times 10^{-2}$	35 / 1599
	Regulation of defense response to virus	4.36 $\times 10^{-2}$	6 / 71
	Regulation of immune effector process	4.42 $\times 10^{-2}$	14 / 409

† The number of genes in the overlap / the total number of genes in the GO category
