# Supplementary material for: Learning gene networks underlying clinical phenotypes using SNP perturbation
Source: PLoS Comput Biol. 2020 Oct 23;16(10):e1007940. doi: 10.1371/journal.pcbi.1007940 (PMC7584257; doi:10.1371/journal.pcbi.1007940)
Supplement: S3 Table — (PDF) [file pcbi.1007940.s013.pdf]

Phenotype Name	Phenotype Group	Description
PREFVCPP	lung	Baseline FVC % predicted
HPRFVCPP	lung	Hankinson pre BD FVC % predicted
HPRFVCPC	lung	Hankinson pre BD FVC percentile
HPRFVCZS	lung	Hankinson pre BD FVC z-score
PREFEVPP	lung	Pre BD FEV % predicted
HPRFEVPP	lung	Hankinson pre BD FEV % predicted
HPRFEVPC	lung	Hankinson pre BD FEV percentile
HPRFEVZS	lung	Hankinson pre BD FEV z-score
PO1FEVPP	lung	1st Post BD FEV % predicted
HPO1FEPP	lung	1st Hankinson post BD FEV % predicted
HPO1FEPC	lung	1st Hankinson post BD FEV percentile
HPO1FEZS	lung	1st Hankinson post BD FEV z-score
PREFF	lung	Pre BD FEV/FVC ratio (%)
HPRFFPC	lung	Hankinson pre BD FEV/FVC percentile
HPRFFZS	lung	Hankinson pre BD FEV/FVC z-score
MC928	lung	Baseline (pre-diluent) FEV1/FVC ratio
PREFVC	lung	Pre BD FVC
PREFEV	lung	Pre BD FEV
PO1FEV	lung	1st Post BD FEV
MC935b	lung	FEV1 15 minutes after 2puffs albuterol
PREPF	lung	Pre BD peak flow
POSPF	lung	Post BD peak flow
lnpc20	lung	Airway responsiveness to methacholine
rescueBD7day	lung	Rescue BD use last 7 days
preventBD7day	lung	Preventative BD use last 7 days
HEMOG	blood	Hemoglobin
WBC	blood	white blood cell count
MONOPCT	blood	Monocytes %
NEUTPCT	blood	Neutrophils (segs,polys) %
LYMPHPCT	blood	Lymphocytes %
BASOPCT	blood	Basophils %
TOTEOSP	blood	Total eosinophils count by % of WBC
EOSPCT	blood	Eosinophils %
log10ige	blood	Logarithm transformed IgE level
log10eos	blood	Logarithm transformed eosinophil level
