# Supplementary material for: Learning gene networks underlying clinical phenotypes using SNP perturbation
Source: PLoS Comput Biol. 2020 Oct 23;16(10):e1007940. doi: 10.1371/journal.pcbi.1007940 (PMC7584257; doi:10.1371/journal.pcbi.1007940)
Supplement: Attachment — Submitted filename: Responses to Reviewers.pdf [file pcbi.1007940.s014.pdf]

Response to Reviewers' Comments

We would like to thank the reviewers for the insightful comments that helped us improve our paper. Below is our response to the reviewers' comments.

Reviewer 1

Reviewer #1: This paper introduces a method called PerturbNet, which can integrate SNP, gene expression and trait information in a single graphical model that can be used to infer the gene network mediated effect of SNPs on the traits. Furthermore, it can decompose the impact of different SNPs on different network components and also infer the underlying network. The model is based on a sparse Conditional Gaussian graphical model (sCGGM). The authors also introduce a new algorithm for learning the scGGM, which is significantly faster than the current implementations. They apply their approach to simulated data and also to data from the Childhood Asthma Management Program (CAMP) and also compare to other methods such PrediXcan, eCAVIAR and a two-layered LASSO and show that their method is much better for identifying the relevant snps. Overall, this is an interesting piece of work and the PerturbNet method should be helpful to analyze datasets from consortia where all three types of measurements are being made. My only main comments to improve the work are (a) a better description of the method (b) comparisons to related work, (c) availability of PerturbNet. Details follow:

COMMENT

1. The PerturbNet approach is based on a sparse CGGM model, which aims to model p(y,z|x) as p(y|x)p(z|y) where x is the SNP information, y is the expression and z is the phenotypic traits. However, fig 1 does not provide any description about the components of the sCGGM approach that is being used. A more annotated version of fig 1 which introduces the different components of the model would be very helpful to the reader. The manuscript text could also go into more details of the methods rather than pushing everything to methods.

RESPONSE

As the reviewer suggested, we annotated Fig 1 and moved a large part of the text on the details of the PerturbNet method from Supporting Information to the main text on pages 12-15.

COMMENT

2. The simulation makes use of a GGM and CGGM model and hence it is not surprising that PerturbNet works very well here. It might be good to discuss the simulation model in the content of the modeling assumptions of the different methods.

RESPONSE

We would like to clarify that our simulation results were obtained not only from GGM and CGGM but also from linear regression models that are typically used in most other existing methods including PrediXcan and eCAVIAR. In the simulation results in Fig 2, only the ones in the left column were obtained from a GGM and CGGM, but the ones in the right column were obtained from the conventional linear regression models with independent noise models for each phenotype. This is also discussed on page 15 in the text highlighted in red.

COMMENT

3. All the methods compared for linking SNPs to traits via networks are not network based. There have been other pieces of work that aim to identify subnetworks that are perturbed or explain the impact of the SNPs on phenotypes by a network. For example, the work from https://www.ncbi.nlm.nih.gov/pmc/articles/PMC4835676/, https://www.ncbi.nlm.nih.gov/pubmed/25781485, https://www.ncbi.nlm.nih.gov/pubmed/21390271. Comparison of PerturbNet to some network-based methods would be very beneficial.

RESPONSE

Thank you for pointing out the additional previous work. We would like to clarify that unlike PerturbNet, these existing methods do not learn the gene network, along with genetic effects on traits; thus, they are not directly comparable to PerturbNet. The existing methods either assumed a known network (Kim et al., 2011), or looked for correlated associations only in a post processing step after performing single-SNP/single-trait associations (Botzman et al., 2016; Oren et al., 2015). In contrast, our method simultaneously learns both the gene network perturbed by SNPs and genetic effects on traits mediated by this network. In order to make the relationship between our work and these existing methods clear, we added the citations to the papers that the reviewer pointed out and a discussion on these on page 2.

COMMENT

4. The module identification step is not discussed in much detail. It is unclear how the number of modules is defined, why it is needed and if and how the module information is leveraged for network inference. It seems that it is used primarily as a post-processing step. It is also not clear how the modules are defined, is it on the network structure inferred or is it on the precision matrix or some other weighted graph.

RESPONSE

Yes, the module identification is used as a post-processing step after the PerturbNet model estimation, and is not a part of the network inference process in PerturbNet. The module identification is done on the network Lambda_y in the estimated PerturbNet model. We added details on the module identification on page 5.

COMMENT

5. The number of samples in the dataset seems low. It is unclear if the authors can assert any confidence on the inferred model. The FDR is mentioned but it is unclear how it is estimated.

RESPONSE

In PerturbNet, false discovery rate, computed as (# of non-zero elements in the estimated model AND in the true model)/(# of non-zero elements in the estimated model), can be obtained only when the ground-truth is known as in simulation study. However, to address the reviewer's concern on the small sample size and confidence on the inferred model, as Reviewer 2 suggested, we performed a robustness analysis with the CAMP datasets modified with fewer samples or with noise added to the expression and clinical trait data. We found our estimates are generally robust with small perturbation of the data. These results are included in the text on page 7 and Fig 7.

COMMENT

6. The code for PerturbNet is not available. This is important to make available as soon as possible.

RESPONSE

The PerturbNet software is available at https://github.com/sssykim/PerturbNet.

COMMENT

7. The Gaussian assumption has some nice properties which make the inference tractable. But it might be worth discussing if the variables, namely the traits are Gaussian.

RESPONSE

As the reviewer suggested, we verified the marginal distribution of each individual trait generally follows univariate Gaussian distribution. We added the QQ-plots to support this observation in S7 Fig and a discussion on the roughly Gaussian-distributed clinical phenotypes on page 16.

COMMENT

8. The new Mega-sCGGM method seems a nice contribution of this work. Code for this should be made available. It would also be helpful to see if this more efficient version has the same or different performance as the original/existing implementations of CGMMs.

RESPONSE

We made the code available on gibhub (see our response to the comment on code availability above). We added text on page 9 to clarify that the previous method NCD as well as Fast-sCGGM and Mega-sCGGM reach the same solution, as they solve the same optimization problem that is convex with unique optimum. We added text on page 4 to clarify that we observed this in our experiments.

COMMENT

9. I might have missed this, but there must be hyperparameters to control for sparsity, but I did not see how this was set.

RESPONSE

We used Bayesian information criterion (BIC) to select the regularization parameters. We added this in sections “Comparison of methods on simulated data” on page 16 and “comparison of methods on asthma data” on page 17.

COMMENT

Minor:
1. Please define CLPP on page 12

RESPONSE

We added the definition of CLPP on page 16.

COMMENT

2. The number of modules in the simulation is not clear. The sentence "..were assumed to have modules of size 50 each, leading to 100 and 2 modules in each network." needs revision (line 442).

RESPONSE

We revised the text to make the network and module size clear on page 15.

Reviewer 2

McCarter et al. focus on an important research problem of identifying SNPs that influence phenotypes via molecular networks. While a number of computational methods exist for various GWAS and eQTL analysis, very few methods, if any, exist that connect genotype to phenotypes via transcriptional networks.

Authors propose a model that builds on probabilistic graphical models, in particular (multivariate) conditional Gaussian graphical model (or Markov random field). The model is constructed as a chain-structured graph in such a way that transcriptional level (or network) is conditioned with genotype and phenotype level is conditioned by the transcriptional networks. This construction allows considering perturbations in the genotype to affect phenotypes via the intermediate transcriptional networks. Authors propose a sparsity promoting optimisation method for the model including all layers using the standard L1 regularizers. Authors also propose three inference procedures to study the learned model to gain more detailed understanding of how genotype affects phenotypes via transcriptional networks. The model is coherently defined in the probabilistic graphical modeling framework. The model is well-motivated, although it is built on the assumption of all variables having a joint Gaussian distribution. Despite the joint normal assumption, the method can reveal reasonable results and probably serves as a good first approximation. The model and methods are clearly described and seems technically correct. Results demonstrate good performance relative to the state-of-the-art.

COMMENT

Major comments:

- page 9: The model space is extremely large while only limited amounts of data is typically available. Although the model learning relies on standard regularisation methods, I am worried that the model learning can be very sensitive. Authors should demonstrate the robustness (or sensitivity) or they model learning with an appropriate robustness analysis. E.g. how much does the learned model/network structure differ for small perturbations in the input data? How much do these changes affect the interpretation of the model (ref. the proposed 3 inference methods)? Are the results in Results section sensitive to small perturbations in the training data? I am OK with the method being marginally sensitive, as long as authors give a comprehensive evaluation of their methods, and give a fair discussion of potential limitations as well.

RESPONSE

As the reviewer suggested, we performed a robustness analysis (text on page 7 and Fig 7). With the CAMP datasets modified with fewer samples or with noise added to the expression and clinical trait data, we found our estimates are generally robust with small perturbation of the data.

COMMENT

- page 12: For simulation studies, data is generated from Gaussian distributions. Can you carry out simulated experiments where data is generated from a model that includes nonlinearities? Authors should describe how realistic the simulation set-up is? Or mention that data is simulated from a model that does not necessarily represent a realistic system.

RESPONSE

In our simulation studies, we used both our own models (GGM/CGGM) (Fig 2 left column) and linear regression models (Fig 2 right column) that have been widely adopted in the literature for GWAS and eQTL mapping. Thus, we feel it is beyond the scope of our paper to investigate the effects of non-linearities on the performance of various models. However, we agree that this is future work that is clearly worth exploring, so we added discussion on how our model might be extended to model non-linearity in the Discussion section on page 8.

COMMENT

Minor comments:

Page 7, row 282: A reader can easily misinterpret that x is a three-valued vector of length p, but text says "given as minor allele frequencies". Can you define the domain of x similarly as you do for y and z.

RESPONSE

We revised the text on page 8, as the reviewer suggested.

COMMENT

Page 9, row 331: I understood that the speed improvement (of several orders of magnitude) is based on *empirical* analysis. Please explicitly clarify that in the text too.

RESPONSE

Yes, we made this clarification on page 12.

COMMENT

pages 10-11: Could authors specify and write the joint Gaussian distribution for the (whole) model from which the marginals (e.g. Eq. On row 381-382) could be obtained using standard marginalisation techniques?

RESPONSE

We added this on page 10 in the text.
